# Joint inference for telemetry and spatial survey data

**DOI:** 10.1002/ecy.4457

**Published:** 2024-10-30

**Authors:** Paul G. Blackwell, Jason Matthiopoulos

**Affiliations:** ^1^ School of Mathematics and Statistics, University of Sheffield Sheffield UK; ^2^ School of Biodiversity, One Health and Veterinary Medicine, University of Glasgow Glasgow UK; ^3^ MacArthur Green Glasgow UK

**Keywords:** data collection effort, Langevin diffusion, Markov chain Monte Carlo, movement models, resource selection functions, step selection functions, transects

## Abstract

Data integration, the joint statistical analysis of data from different observation platforms, is pivotal for data‐hungry disciplines such as spatial ecology. Pooled data types obtained from the same underlying process, analyzed jointly, can improve both precision and accuracy in models of species distributions and species–habitat associations. However, the integration of telemetry and spatial survey data has proved elusive because of the fundamentally different analytical approaches required by these two data types. Here, “spatial survey” denotes a survey that records spatial locations and has no temporal structure, for example, line or point transects but not capture–recapture or telemetry. Step selection functions (SSFs—the canonical framework for telemetry) and habitat selection functions (HSFs—the default approach to spatial surveys) differ in not only their specifications but also their results. By imposing the constraint that microscopic mechanisms (animal movement) must correctly scale up to macroscopic emergence (population distributions), a relationship can be written between SSFs and HSFs, leading to a joint likelihood using both datasets. We implement this approach using maximum likelihood, explore its estimation precision by systematic simulation, and seek to derive broad guidelines for effort allocation in the field. We find that complementarities in spatial coverage and resolution between telemetry and survey data often lead to marked inferential improvements in joint analyses over those using either data type alone. The optimal allocation of effort between the two methods (or the choice between them, if only one can be selected) depends on the overall effort expended and the pattern of environmental heterogeneity. Examining inferential performance over a broad range of scenarios for the relative cost between the two methods, we find that integrated analysis usually offers higher precision. Our methodological work shows how to integrate the analysis of telemetry and spatial survey data under a novel joint likelihood function, using traditional computational methods. Our simulation experiments suggest that even when the relative costs of the two methods favor the deployment of one field approach over another, their joint use is broadly preferable. Therefore, joint analysis of the two key methods used in spatial ecology is not only possible but also computationally efficient and statistically more powerful.

## INTRODUCTION

There is a wide variety of technologies and field protocols for collecting spatial data on the distribution of animals. However, the majority of the resulting data fall into one of two broad classes (Matthiopoulos et al., 2023), either telemetry (radiotelemetry, satellite tracking, geolocators, archival tags; Cagnacci et al., 2010) or spatial surveys (line transects, strip transects, point transects, grid counts; Buckland et al., 2005).

Here, and throughout the paper, we use “spatial survey” as a shorthand for an observation process that records locations in space and that has no temporal structure. It may represent observation at a single time giving a partial snapshot of the locations of individuals, or observations at multiple fixed times provided that they are sufficiently separated in time that, given the animals' long‐term distribution, there is no temporal dependence between the different times. So our usage includes, for example, line or point transects but not capture–recapture—spatial or otherwise—or telemetry. (We will sometimes simply refer to “survey” data where no ambiguity results.) There is a clear conceptual divide between these two data types. Spatial surveys focus on particular regions of space and can in principle observe any individual from the population that comes into detection range. In contrast, telemetry studies focus on particular individuals and can in principle observe any region in space visited by the tagged animals. Analytically, the two data types correspond to two different ways of thinking about spatial processes (Phillips et al., 2019; Turchin, 1998). The Lagrangian viewpoint, which best aligns with telemetry data, considers trajectories through space–time and often gives rise to microscale models of individual movement. The Eulerian perspective, which has more affinity with spatial survey data, considers the density of utilization of any given point in space and often gives rise to macroscale models of population distribution. The links between these formalisms are a fruitful area of research in spatial ecology, looking at how small‐scale processes of movement give rise to spatial heterogeneity in large‐scale usage (Moorcroft & Lewis, 2006; Okubo, 1980; Turchin, 1998).

Ostensibly, both of these observation platforms are extracting data from the same underlying biological processes (habitat preferences and spatial abundance), which form the focus of our statistical inference. Therefore, despite their fundamental differences in perspective, both telemetry and spatial survey data have been used in the past to derive species distribution maps (e.g., compare Matthiopoulos et al. (2004) and Herr et al. (2009) using telemetry and surveys, respectively, to model the distribution of the same species) and to model species–habitat associations (e.g., telemetry: Aarts et al., 2008; survey: Hedley & Buckland, 2004).

As datasets from spatial surveys that coincide or overlap with tagging projects are increasingly being stored and visualized on common software platforms (Fujioka et al., 2014; Marvin et al., 2016), it seems opportune to combine these datasets quantitatively, not only to increase the effective sample size of the resulting data, but also to explore whether the datasets have complementary inferential value (González‐Solís & Shaffer, 2009).

Early work on combining telemetry with spatial survey data made the plausible assumption that the results obtained from analyzing telemetry and survey data should agree. Therefore, some papers in this area (Ball et al., 2005; Pinto et al., 2016; Prichard et al., 2019) have used one datatype for validation of the results of the analysis of the other type.

Other studies have exploited the different information carried by spatial survey and telemetry data for purposes of calibration. For example, Matthiopoulos et al. (2004) and Jones et al. (2015) use survey data to constrain the utilization distributions generated by telemetry analysis; Bächler and Liechti (2007), Udevitz et al. (2008), Popescu et al. (2017), Willson et al. (2018), and Boback et al. (2020) use telemetry to ground‐truth the absolute detection probabilities of surveys; Louzao et al. (2009) and Camphuysen et al. (2012) use telemetry to generate foraging‐specific distributions from survey data; and Yamamoto et al. (2015) try to understand the composition of survey maps in terms of population components observed by tracking.

The above efforts are all good examples of the complementary use of telemetry and spatial surveys, recognizing that their joint inferential power goes beyond a simple increase in effective sample size. Hence, rather than thinking of data integration solely as a route to improve precision, we are also recognizing it as a route for correcting bias. More broadly within ecology, the notion of complementarity in data integration is making a remarkable impact on how traditional analyses are viewed, leading to the re‐examination of problems of bias for different types of data (Fletcher et al., 2016, 2019; Matthiopoulos et al., 2022; Miller et al., 2019; Nelli et al., 2019; Pacifici et al., 2017; Reich et al., 2018).

However, full integration between telemetry and spatial survey data has not yet been possible without severe information losses. The few studies (Louzao et al., 2009; Pikesley et al., 2018) that have attempted a joint analysis have tended to use purely graphical methods or telemetry censoring and abundance thresholding to convert the data into a similar form, amenable to the same likelihood. A major obstacle to joint inference is the incongruence between the statistical frameworks used for these two data types. Telemetry data are most conveniently analyzed via step selection functions (SSFs; Thurfjell et al., 2014), while resource or habitat selection functions (HSFs; Boyce et al., 1999) are most appropriate for survey data. These approaches do not, by default, lead to the same results. Specifically, scaling up by simulation the microscopic model obtained via SSFs does not yield the same steady‐state distribution generated by an HSF (Barnett & Moorcroft, 2008; Moorcroft & Barnett, 2008; Signer et al., 2017).

A promising development in this area is the convergence between the frameworks of resource selection and step selection analyses both in discrete time (Michelot, Blackwell, Chamaillé‐Jammes, et al., 2019; Michelot, Blackwell, & Matthiopoulos, 2019) and in continuous time using a Langevin diffusion (Michelot, Gloaguen, et al., 2019). This work has established the conditions under which SSF and HSF frameworks agree, and has derived methods for HSF‐type inference from telemetry (Michelot, Blackwell, Chamaillé‐Jammes, et al., 2019; Michelot, Blackwell, & Matthiopoulos, 2019; Michelot, Gloaguen, et al., 2019). These new methods rely on implicit assumptions of representativeness. In particular, they require that the telemetry tagged individuals are randomly and cross‐sectionally selected from the same population mapped by the spatial survey methods. They also require that any aspects of selection that are not modeled explicitly in terms of covariates—that is, aspects subsumed in the stochastic terms of the model—do not vary systematically between the environment within the survey study area and the environment explored by the spatially unconstrained tagged individuals. Of course, such assumptions about the limited effects of unmodeled structure are pervasive, perhaps inevitable, in modeling, and certainly far from unique to that approach.

Related work concerns the incorporation of telemetry data into spatial capture–recapture (SCR) modeling; typically, the implicit spatial modeling that underlies SCR is much simpler than is used in the analysis of telemetry, but McClintock et al. (2022) give a review and discussion of recent developments. For example, Chandler et al. (2022) use an Ornstein–Uhlenbeck (OU) process (see *Localization using the OU process*) to model the underlying movement and to accommodate autocorrelation in detection probabilities. Hostetter et al. (2022) avoid the usual assumption of stationary home ranges by modeling capture probabilities conditionally on random walk movement models, incorporating covariate information, though their distributions for initial locations do not use the covariate information in a way that is coherent with the movement modeling. Bassing et al. (2022) use telemetry data only as “used” locations in an HSF, for comparison with occupancy modeling, and so do not allow for the autocorrelation and local dynamics of movement. Perhaps the SCR approach most closely related to our work here is that of Gardner et al. (2022), who use a discrete‐time version of the Langevin process of Michelot, Gloaguen, et al. (2019) to represent both movement and initial expected locations in a coherent way. Their simulations discretize the initial spatial distributions, and include cases with SCR data only and with auxiliary telemetry data.

Glennie et al. (2021) use movement data in a different way to augment data arising from distance sampling, when the assumption that the sampling is effectively instantaneous is not tenable. They assume that individuals follow a relatively simple movement model (Brownian motion) and that an individual's probability of detection in the distance sampling depends on the whole of its path during the time of the sampling. They then use telemetry data that are independent of the distance sampling and jointly estimate the movement and detection parameters, correcting the interpretation of the distance sampling data to allow for movement during the survey.

Here, we focus on developing an expandable analytical framework for joint analysis of telemetry and spatial survey data when the spatiotemporal frames and population members observed by survey are representative of those observed by telemetry, and vice versa. We investigate the quality of inference in different scenarios of data availability and thus derive insights on how effective sample size and complementarity work in this setting. In our *Discussion*, we revisit some of our assumptions and consider potential avenues for relaxing them.

## ANALYSIS FRAMEWORK

### Modeling philosophy

The motivation for the approach adopted by Michelot, Blackwell, and Matthiopoulos (2019) and Michelot, Blackwell, Chamaillé‐Jammes, et al. (2019) originates from computational methods for statistical inference, and in particular, the broad class of Markov chain Monte Carlo algorithms (Hastings, 1970). Computational inference methods often involve a procedure in which a search particle moves through parameter space, responding to density gradients (density is usually either the posterior probability density in Bayesian approaches or the normalized likelihood in frequentist approaches, but might also cover other quantities such as entropy in machine learning algorithms [Phillips et al., 2006] or fitness in genetic algorithms [Barricelli, 1957]). Unlike maximum likelihood algorithms, which perform optimization, MCMC does not prioritize searching for the point of peak density (the mode, or maximum likelihood point), but, rather, tries to faithfully approximate the entire *density landscape*. It achieves this by adopting a search pattern that is guaranteed to visit locations in parameter space with a relative frequency proportional to their underlying density. The distribution of such visits thus gives an approximation of the underlying density landscape. Therefore, the derivation of MCMC algorithms prizes the property that individual (microscopic) particles describe with their movement an underlying steady‐state (macroscopic) distribution. Borrowing the properties of these algorithms for specifying the rules of movement for SSFs is therefore guaranteed to give us steady‐state distributions, the surfaces of population space‐use (utilization) described by HSFs. Imposing this requirement on the two inferential frameworks of SSF and HSF leads to a tractable mathematical relationship between their selection coefficients. Such a relationship can allow us to conduct joint inference of telemetry and spatial survey data because they are essentially being used to estimate only one set of coefficients.

A key challenge of this approach therefore is to formulate movement models for use by the SSF framework that maintain the essential MCMC scaling properties, while at the same time being realistic models for animal movement. It transpires that this class of models is sufficiently broad to cover many of the movement models commonly used in the ecological literature. A minimally realistic model for movement in SSFs must encompass stochasticity (e.g., diffusive movement such as a random walk) and should allow for the existence of a central point of attraction (advective movement toward, e.g., a nest or colony) and of environmental gradient climbing (advective movement toward locations of higher habitat suitability). It should also be defined in continuous time since the arbitrary (and often irregular) sampling intervals of telemetry studies are not necessarily the most appropriate scales for defining movement processes. We describe such a minimally realistic movement model in the following section.

### Movement modeling

We envisage an underlying utilization distribution $\pi \left(x\right)$ which emerges from the long‐term use of space by multiple moving individuals. The model describing the movement of each individual that satisfies our minimally realistic requirements, above, is a Langevin diffusion (see Michelot, Gloaguen, et al., 2019 for its original application to animal movement), a modification of Brownian motion. Like the Ornstein–Uhlenbeck (OU) process (Uhlenbeck & Ornstein, 1930), better known in movement modeling (Blackwell, 1997; Dunn & Gipson, 1977) and discussed in *Localization using the OU process*, the Langevin diffusion adds a *drift* (i.e., expected movement) term to the random movement of Brownian motion; in contrast with the OU process, in the Langevin case, the drift term can vary flexibly from place to place. Specifically, the form of the drift term is related to the utilization distribution, giving rise to the relationship between short‐term and long‐term distributions.

More precisely, the Langevin diffusion for location x satisfies the stochastic differential equation (e.g., Klebaner, 2012; Øksendal, 2010)

(1) $$dx=b\left(x\left(t\right)\right)\mathrm{dt}+\sqrt{\Gamma }dW\left(t\right),$$

where $W\left(t\right)$ is a two‐dimensional Brownian motion, Γ controls the speed of the process, and

(2) $$b\left(x\left(t\right)\right)=\frac{\Gamma }{2}\nabla \log\left(\pi \left(x\left(t\right)\right)\right),$$

is the drift term related to the utilization distribution $\pi \left(x\right)$. The operator $\nabla \left(\cdot \right)$ represents the vector gradient of a surface.

In practice, we work with a time‐discretization of this process. The standard Euler–Maruyama approach approximates the process over a short time step δt by

(3) $$x\left(t+\mathrm{\delta t}\right)=x\left(t\right)+b\left(x\left(t\right)\right)\mathrm{\delta t}+\epsilon ,\;\epsilon ∼N\left(0,\Gamma \mathrm{\delta t}I\right),$$

where I is the 2×2 identity matrix. The key mathematical properties of the process hold exactly only in the continuous‐time case (i.e., in the limit as δt→0). For simulation, we can choose δt to be small compared with the timescale of the observations; details are given in *Simulations*. For inference, Michelot, Gloaguen, et al. (2019) show in the telemetry‐only case that this approximation, with δt the interval between observations, can enable us to make inference about selection parameters, provided the interval between observations is not too long. However, here we use an improved estimation approach, as discussed in the next section.

### Movement likelihood

We assume that telemetry data $x_{0},\ldots ,x_{n}$ are collected sequentially at times $t_{0},\ldots ,t_{n}$, and write $\delta t_{j}$ for $t_{j+1}-t_{j}$. Often, all $\delta t_{j}s$ will be equal, but because of the continuous‐time formulation that is not required.

To carry out inference for the movement model of *Movement modeling*, one possibility is to apply directly the approximation of Equation (3), as in Michelot, Gloaguen, et al. (2019). Each observation $x_{1},\ldots ,x_{n}$ has a normal distribution that depends on the previous one, and as is typical in movement analysis we assume that the initial location $x_{0}$ carries no relevant information, and simply condition on it (see the *Discussion* for alternatives). The overall likelihood from the movement data is therefore

(4) $$l_{M}\left(\beta ,\Gamma \right)=\prod_{j=0}^{n-1}\phi \left(x_{j+1}|x_{j}+b\left(x_{j}\right)\delta t_{j},\Gamma \delta t_{j}I\right),$$

where β denotes the parameters of the utilization distribution $\pi \left(\cdot \right)$ and $\phi \left(x|\mu ,\sum \right)$ denotes the density at x of the bivariate normal distribution with mean vector μ and covariance matrix ∑, giving the log‐likelihood

(5) $$L_{M}\left(\beta ,\Gamma \right)=\sum_{j=0}^{n-1}\log\phi \left(x_{j+1}|x_{j}+b\left(x_{j}\right)\delta t_{j},\Gamma \delta t_{j}I\right).$$

Note that if the utilization distribution $\pi \left(\cdot \right)$ is flat, then $b\left(\cdot \right)\equiv 0$ and the movement process is just Brownian motion.

Michelot, Gloaguen, et al. (2019) show, however, that this relatively simple approach to inference from the Langevin diffusion is susceptible to bias. This potential for bias is a limitation of the inference algorithm, not inherent in the movement data. It arises because the discretization in Equation (3) depends on $b\left(\cdot \right)$ being approximately constant along the animal's path between observations. If $b\left(\cdot \right)$ were constant over the path from $x_{j}$ to $x_{j+1}$, then the likelihood contribution from $x_{j+1}|x_{j}$ would be exactly as implied by Equation (3); there would be no approximation, and no possibility of bias. The bias therefore depends in a complex way on the rate of change of $b\left(.\right)$ along the individual's possible paths, relative to the interval between observations, and thus on the speed of movement as determined by the diffusion rate Γ and the drift term $b\left(.\right)$ itself, on the interval between observations δt, and on the spatial variation in $b\left(.\right)$, that is the rate of curvature in the logarithm of the utilization distribution $\pi \left(\cdot \right)$. More frequent observations, a lower diffusion rate, and a utilization distribution that is slowly varying will all tend to lead to lower bias, but in general, these cannot be guaranteed. Approaches that involve reconstructing the movement trajectories in detail, for example, Parton et al. (2017), would be more accurate, because they could use information about the drift term $b\left(x\right)$ along the trajectory, but they are computationally infeasible for large datasets. Instead, here we modify the approximation of Equation (3) in a way that can be computationally cheap but which reduces the bias effectively. Instead of using the drift term $b\left(x_{j}\right)$ defined as a function of the gradient at $x_{j}$, as in Equation (2), we use the corresponding drift integrated over points (here denoted by z) in a neighborhood of $x_{j}$; more precisely, we define

(6) $$\tilde{b}\left(x_{j}\right)=\frac{\Gamma }{2}\int \nabla \log\left(\pi \left(z\right)\right)\mathrm{dG}\;\left(z|x_{j},\zeta \sqrt{\delta t_{j}}\;\right),$$

where $G\;\left(\cdot |x_{j},\zeta \sqrt{\delta t_{j}}\;\right)$ represents a symmetric distribution centered at $x_{j}$ with scale parameter $\zeta \sqrt{\delta t_{j}}$. Choosing the scale parameter to be comparable with the distance moved in time $\delta t_{j}$ means that the gradient term used matches more closely the true gradient of the process over that time interval. Note that in the limit for small $\delta t_{j}$, this is equivalent to using Equation (2). Theoretically, $G\left(\cdot \right)$ would be a continuous distribution, with density $g\left(\cdot \right)$ say so that

(7) $$\tilde{b}\left(x_{j}\right)=\frac{\Gamma }{2}\int g\;\left(z|x_{j},\zeta \sqrt{\delta t_{j}}\;\right)\nabla \log\left(\pi \left(z\right)\right)dz,$$

but empirically, even a very simple discrete choice for $G\left(\cdot \right)$ can address the issue of bias; see *Inference* for an example of the choice of $G\left(\cdot \right)$ and of ζ. It is important to note that $G\left(\cdot \right)$ and ζ are not part of the model, and so do not have biological meanings; they simply give a mechanism for more accurate fitting of the model in Equation (1). Specifically, replacing $b\left(\cdot \right)$ with $\tilde{b}\left(\cdot \right)$ in Equation (3) improves that approximation, as used for inference, but the underlying “true” continuous‐time model is still defined in terms of the original $b\left(\cdot \right)$ in Equation (1), and the utilization distribution is unchanged and given by $\pi \left(\cdot \right)$ (e.g., as in Equation 9 below).

### Environment model

As is widespread in modeling species distributions (Matthiopoulos et al., 2023), we take the utilization distribution for all individuals to be proportional to the exponential of a linear predictor $\eta \left(x\right)$ written as a linear combination of spatially varying covariates $c_{i}\left(\cdot \right)$:

(8) $$\pi \left(x\right)\propto \exp\left(\eta \left(x\right)\right)$$

(9) $$=\exp\left(\beta_{1}c_{1}\left(x\right)+\cdots +\beta_{k}c_{k}\left(x\right)\right).$$

Formally, each $c_{i}\left(\cdot \right)$ is a smooth (differentiable) function over continuous space. In practice, each $c_{i}\left(\cdot \right)$ is likely to be a smoothed version of a grid of observed values; see *Simulations* for details. These results can be generalized to include higher order terms of the covariates, as well as interaction terms between them.

With this utilization distribution, the Langevin drift term is

(10) $$b\left(x\right)=\frac{\Gamma }{2}\sum_{i=1}^{k}\beta_{i}\nabla c_{i}\left(x\right),$$

and the improved approximation of Equation (6) is

(11) $$\tilde{b}\left(x\right)=\frac{\Gamma }{2}\sum_{i=1}^{k}\left\{\beta_{i}\int \nabla c_{i}\left(z\right)\mathrm{dG}\;\left(z|x,\zeta \sqrt{\delta t_{j}}\;\right)\right\}.$$

### Spatial survey model

We take the simplest possible model of the survey process: an instantaneous “snapshot” over a particular region, A, which may represent a single connected area, or a collection of strips or neighborhoods of points. Each individual in A at the survey time has some common probability of being observed. Observations therefore form an inhomogeneous Poisson process (IPP) with intensity

(12) $$\lambda \left(x\right)\propto \pi \left(x\right).$$

For applications of IPP for species distribution models, see Matthiopoulos et al. (2023). We can write

(13) $$\lambda \left(x\right)=\exp\left(\alpha +\eta \left(x\right)\right),$$

where the intercept α will depend on population size, detectability, and survey effort.

If observed locations are $y_{1},\ldots ,y_{m}$, then the likelihood from the survey data is

(14) $$l_{s}\left(\alpha ,\beta \right)=\exp\left(-\int_{A}\lambda \left(y\right)dy\right)\prod_{i=1}^{m}\lambda \left(y_{i}\right)$$

(15) $$=\exp\left(-\int_{A}\exp\left(\alpha +\eta \left(y\right)\right)dy\right)\prod_{i=1}^{m}\exp\left(\alpha +\eta \left(y_{i}\right)\right)$$

(16) $$=\exp\left(-\exp\left(\alpha \right)\int_{A}\exp\left(\eta \left(y\right)\right)dy\right)\exp\left(m\alpha \right)\prod_{i=1}^{m}\exp\left(\eta \left(y_{i}\right)\right).$$

The log‐likelihood is therefore

(17) $$L_{s}\left(\alpha ,\beta \right)=-\exp\left(\alpha \right)\int_{A}\exp\left(\eta \left(y\right)\right)dy\;+m\alpha +\sum_{i=1}^{m}\eta \left(y_{i}\right).$$

### Overall likelihood

The diffusivity parameter Γ scales the variance in Equation (3); for convenience, we work with $\gamma =\log\left(\Gamma \right)$. The combined log‐likelihood is

(18) $$\begin{array}{l} L\left(\alpha ,\beta ,\gamma \right)=L_{s}\left(\alpha ,\beta \right)+L_{M}\left(\beta ,\gamma \right)\; \end{array}$$

(19) $$=-\exp\left(\alpha \right)\int_{A}\exp\left(\eta \left(y\right)\right)dy\;+m\alpha +\sum_{i=1}^{m}\eta \left(y_{i}\right)\;+\sum_{j=0}^{n-1}\log\phi \left(x_{j+1}|x_{j}+\tilde{b}\left(x_{j}\right)\delta t_{j},\exp\left(\gamma \right)\delta t_{j}I\right),$$

and is a function of three groups of parameters: $\beta =\beta_{1},\ldots ,\beta_{k}$, which define resource selection; γ, which relates to speed of movement; and α, which relates to effective population size, observability, and survey effort.

Strictly, $\tilde{b}\left(\cdot \right)$ and $\eta \left(\cdot \right)$ depend on the parameters too, so more precisely we should write the combined log‐likelihood as

(20) $$\begin{array}{l} L\left(\alpha ,\beta ,\gamma \right)=-\exp\left(\alpha \right)\int_{A}\exp\left(\eta \left(y,\beta \right)\right)dy\;+m\alpha +\sum_{i=1}^{m}\eta \left(y_{i},\beta \right)\\ \;+\sum_{j=0}^{n-1}\log\phi \left(x_{j+1}|x_{j}+\tilde{b}\left(x_{j},\beta ,\gamma \right)\delta t_{j},\exp\left(\gamma \right)\delta t_{j}I\right). \end{array}$$

The function $\tilde{b}\left(\cdot \right)$ also depends on $G\left(\cdot \right)$ and ζ, but these relate to the discretization approximation rather than having any biological meaning, and are fixed empirically to optimize the approximation, rather than being formally estimated, so are omitted here.

For abundance estimation, the intercept parameter α would be of central importance. Studies of animal mobility might focus on the diffusivity parameter γ. However, the key application of this framework of joint inference is in species–habitat association studies. There, α and γ are nuisance parameters; β affects selection and is the parameter of interest, although in some cases, elements of β may effectively be nuisance parameters too—see *Selection and localization*.

### Selection and localization

For the idea of a utilization distribution to be meaningful as a probability density function, it is necessary for $\pi \left(x\right)$ to integrate to 1, or equivalently for the integral of $\exp\left(\eta \left(x\right)\right)$ to be finite.

One way is for the available area to be finite—an “island” model—and for the movement model to respect this through the so‐called reflecting boundary conditions. Alternatively, to capture “nomadic” behavior, a finite region can be used to represent an unbounded space by using periodic boundary conditions, treating a modeled rectangular region as a torus. A third possibility is an “oasis” model, where covariates representing tangible, desirable resources (i.e., with positive selection coefficients) decrease markedly with distance away from some more desirable area, leading naturally to a finite integral for $\eta \left(x\right)$ over an infinite modeled area. A fourth way is to allow additional covariates, perhaps entirely notional, that are separate from the selected resources of interest and represent a localizing tendency due to memory, social behavior, or some other phenomenon distinct from resource selection, that is, an “attraction” model.

This last approach is used in the simulation examples here; practicalities, and the mathematical details of one specific form of localizing tendency, are discussed in *Localization using the OU process*. This approach has the appealing feature that the additional covariates simply appear as extra terms, with corresponding coefficients $\beta_{i}$, in the same form as those already appearing in the equations defining the utilization distribution (9) and the Langevin drift terms (10 and 11).

In any given analysis, the choice between these approaches is largely one of biologically appropriate modeling of the animals' space use. Where there is a well‐defined natural boundary to the region used, for example, for terrestrial species on an actual island, then it makes sense to use an “island” approach that respects that boundary and needs no other mechanism for localization. Similarly, in a case where space use is limited through the spatial distribution of measurable covariates, the “oasis” model applies naturally, and no explicit localization is needed (except perhaps to constrain the sign of one or more coefficients). Alternatively, if no such covariate is available, but the localization is thought to represent a preference for being near a central place, or just a general preference for familiar places, that suggests an “attraction” model as detailed in the examples below. If the previous cases do not apply, then the “nomadic” approach gives a way of ensuring that the necessary integral is finite, provided covariate information is available over a sufficiently large region. All these cases would be straightforward to include in the current framework, since they require only minor changes to boundary conditions or construction of “latent” covariates (as detailed in *Localization using the OU process*). If none of these apply, that would typically suggest that the movement modeling needs to take into account the values of covariates in locations that are not observed, which is problematic for any inference framework, not just the one proposed here. An exception would be where movement and covariates are at a large enough scale that it is appropriate to use the whole of Earth as the space over which distribution is defined, for example, long‐range movement by marine mammals (Brillinger & Stewart, 1998); in that case, the conceptual framework still applies, but some adjustment is necessary to take into account the appropriate spherical geometry (Brillinger et al., 2002; Brillinger & Stewart, 1998).

#### Localization using the OU process

The OU process is a simple diffusion process with a stationary distribution, and therefore a special case of the Langevin diffusion; its stationary or utilization distribution is just a bivariate normal distribution. As such, it is a convenient way of addressing the “localization” issue raised in *Selection and localization*. It may also serve as an illustrative example or building block for representing habitat selection more generally.

As an aside, the OU process is unique in that Equation (3) holds exactly; steps within a purely OU movement model follow a normal distribution without any approximation. This is important when the OU process is used as a movement model in its own right (Blackwell, 1997, 2003; Chandler et al., 2022; Dunn & Gipson, 1977) but less useful here, since we are mainly interested in combining it with less tractable elements.

A general OU process has

(21) $$\pi \left(x\right)=\phi \left(x|\mu ,\Lambda \right)$$

(22) $$\propto \exp\left(-\frac{1}{2}{\left(x-\mu \right)}^{T}\Lambda^{-1}\left(x-\mu \right)\right).$$

Here, we consider only the circular case, Λ=λI, with λ>0. Writing $x=\left(x,y\right)$ and $\mu =\left(\mu_{x},\mu_{y}\right)$, we have

(23) $${\left(x-\mu \right)}^{T}\Lambda^{-1}\left(x-\mu \right)=\lambda^{-1}{\left(x-\mu \right)}^{T}\left(x-\mu \right)$$

(24) $$=\lambda^{-1}\left({\left(x-\mu_{x}\right)}^{2}+{\left(y-\mu_{y}\right)}^{2}\right)$$

(25) $$=\lambda^{-1}\left(x^{2}+y^{2}-2\mu_{x}x-2\mu_{y}y+\mu_{x}^{2}+\mu_{y}^{2}\right),$$

and hence, treating $\mu_{x}$ and $\mu_{y}$ as constants,

(26) $$\pi \left(x\right)\propto \exp\left(\lambda^{-1}\left(\mu_{x}x+\mu_{y}y-\frac{1}{2}\left(x^{2}+y^{2}\right)\right)\right),$$

so the OU model can be written as a selection model in the form above, with each $c_{i}\left(\cdot \right)$ a simple function of the coordinates of x. We have

(27) $$\pi \left(x\right)\propto \exp\left(\beta_{1}c_{1}\left(x\right)+\beta_{2}c_{2}\left(x\right)+\beta_{3}c_{3}\left(x\right)\right),$$

where $c_{1}\left(x\right)=x,c_{2}\left(x\right)=y,c_{3}\left(x\right)=-\frac{1}{2}\left(x^{2}+y^{2}\right)$ and $\beta_{1}=\mu_{x}/\lambda ,\beta_{2}=\mu_{y}/\lambda ,\beta_{3}=1/\lambda$. The parameters $\beta_{1}$ and $\beta_{2}$ can take any values; because λ is positive, $\beta_{3}$ must also be positive, and this constraint should be incorporated during estimation.

The general bivariate case for Λ leads to a similar form, with five terms ($x,y,x^{2},y^{2},\mathrm{xy}$). Constraints are needed on Λ to ensure that the process has a well‐defined stationary distribution and that it makes sense biologically as a model that is independent of the coordinate system; for details, see Blackwell (1997).

It is therefore straightforward to add a localization term to the habitat selection model, and estimate its parameters jointly with the selection parameters and nuisance parameters, by simply adding a few terms to $\eta \left(\cdot \right)$. This automatically incorporates the localization terms into the likelihoods from both the movement and the survey data, through their inclusion in $\eta \left(\cdot \right),\pi \left(\cdot \right)$ and $\tilde{b}\left(\cdot \right)$. Like the selection terms, they will correspond to known spatial covariates—in this case, just constructed from the coordinates—and unknown parameters so that the details of the localization do not have to be specified in advance. That is, by including these “polynomial” covariates as well as observed environmental ones, we can readily include the case where the utilization distribution is the product of a resource selection term and a bivariate normal density, and estimate all parameters straightforwardly.

## SIMULATION EXAMPLES

### Simulations

For our examples, we focus on learning about a single selection parameter from combinations of telemetry and spatial survey data. We use an attraction approach based on an OU process to localize the model, in the sense of *Selection and localization*; we therefore have

(28) $$\pi \left(x\right)\propto \exp\left(\beta_{1}c_{1}\left(x\right)+\beta_{2}c_{2}\left(x\right)+\beta_{3}c_{3}\left(x\right)+\beta_{4}c_{4}\left(x\right)\right),$$

where $c_{1}\left(\cdot \right)$ is a known spatial covariate representing a resource with selection coefficient $\beta_{1}$ and $c_{2}\left(\cdot \right),c_{3}\left(\cdot \right),c_{4}\left(\cdot \right)$ are simple known functions of x, with associated nuisance parameters $\beta_{2},\beta_{3},\beta_{4}$.

The covariate $c_{1}\left(\cdot \right)$ is based on a grid of values simulated from a Gaussian random field, using the R package “geoR” (Ribeiro et al., 2022), and then interpolated bilinearly as in Michelot, Gloaguen, et al. (2019) to provide values of $c_{1}\left(\cdot \right)$, and hence $\pi \left(\cdot \right)$, and of the gradient needed by the Langevin movement model.

Given the central tendency built into the model, our simulation is an appropriate model for central‐place foragers, that is, territorial individuals or colonial species. We simulate a colony of individuals, moving independently around the utilization distribution $\pi \left(\cdot \right)$ according to Equation (1), and we observe them in two ways: a number of them are “tagged” and their locations recorded at regular intervals, and a single “snapshot” survey is carried out on a rectangular region, observing those individuals who happen to be in the region at a given time. These observation processes introduce additional parameters α and γ relating to the colony size and the speed of movement, respectively, both treated as unknown nuisance parameters.

In detail, we simulated five resource maps from each of two Gaussian random field models using the exponential covariance function, both with variance parameter 0.1 but with two different values for the spatial range parameter, 0.1 and 0.2, over the region $\left[-1,1\right]\times \left[-1,1\right]$ with grid size 0.005. These two values for spatial range give different levels of spatial autocorrelation in the resource map. Using a selection parameter $\beta_{1}=2.0$, each map was combined with a circular OU attraction model centered at the origin with $\lambda ={\left(0.2\right)}^{2}$ to give a utilization surface that is essentially confined to the simulated region, but where the fine‐scale structure is dominated by the effect of $\beta_{1}$.

On each map, we carried out five replicates of a series of simulation experiments. These involved simulating the movement of a colony of 100 individuals using a fine‐scale approximation to the Langevin movement model using time steps of 0.1, duration 500, and speed parameter $\Gamma =10^{-5}$. Within each replicate, the movement simulation was carried out twice. The first run used starting locations sampled from a bivariate normal distribution, centered at the origin and with SD 0.05 so that the initial locations carried no information about β; thinned to unit time intervals, this run was used to simulate telemetry data with δt=1 from between 5 and 50 individuals. The second run used starting locations sampled from the utilization distribution itself; by taking a “snapshot” of final locations that were inside a square centered at the origin, this run was used to simulate a spatial survey over that region, with the side length of the square varying from 0.1 to 1.0. Strictly speaking, simulating movements to generate the survey data was unnecessary, since we can simulate directly from the utilization distribution, but taking the survey after simulated movement provides reassurance and better comparability.

### Inference

We obtained estimates of all the parameters from the simulated datasets by maximizing the combined likelihood from Equation (20) numerically. This can be interpreted as maximum likelihood estimation or as Bayesian maximum a posteriori (MAP) estimation using a flat prior distribution. The SE/posterior SD was obtained numerically from the Hessian matrix, after maximization. The algorithm used was the “L‐BFGS‐B” quasi‐Newton method with (very wide) bounds based on Byrd et al. (1995), as implemented in “optim” in R Core Team (2021).

To calculate $\tilde{b}\left(x_{j}\right)$, the “locally averaged” gradient term in the movement likelihood, we took a weighted average of $b\left(\cdot \right)$ at $x_{j}$ and at four points

(29) $$x_{j}+\zeta \sqrt{\delta t_{j}}\left(\pm 1_{x}\pm 1_{y}\right),$$

with weights 1/2,1/8,1/8,1/8,1/8, where $1_{x}$ and $1_{y}$ are unit vectors in the x and y directions, respectively, that is at four points equidistant from $x_{j}$ in the diagonal directions defined by the coordinate system. This can be thought of as an exceptionally simple, discrete choice of $G\left(\cdot \right)$ in the integral in (6). A more typical choice would be to take $G\left(\cdot \right)$ to be a circular bivariate normal distribution; nevertheless, our choice largely eliminates the bias in estimation based on the telemetry data in our experiments. In theory, ζ should be related to the movement parameter γ, but instead, we fixed it based on the empirical distances moved, since this means that the additional computational cost over the simpler Langevin algorithm (3) is minimal, and the movement (as opposed to selection) parameters are generally very well estimated. Since the points in (29) are at a distance $\zeta \sqrt{2\delta t_{j}}$ from $x_{j}$, we took

(30) $$\zeta =\sqrt{\frac{1}{2n_{\text{step}}}\sum_{j}\left\{\frac{{\left\|x_{j+1}-x_{j}\right\|}^{2}}{\delta t_{j}}\right\}},$$

where the summation is over all $n_{\text{step}}$ of the steps analyzed so that those distances were on average the same as the distances moved over the same time interval. The spread of points around each location over which the gradient was averaged depended on the duration of the associated step, but not on the length of that individual step or on values of the covariates. For simplicity, the same choice of ζ and $G\left(\cdot \right)$ was used for the OU localization terms as for the selection parameter; the localization terms could be calculated analytically using a different choice of $G\left(\cdot \right)$, but this would complicate the algorithm and its interpretation without any guarantee of improved performance, while any computational gain would be minor in these examples. For other possible choices of ζ and $G\left(\cdot \right)$, see *The effect of* $G\left(\cdot \right)$ and the *Discussion*.

Estimation was carried out for all combinations of the number of telemetry tracks and all sizes of the survey area, plus cases where either no telemetry or no survey was used. This gave a total of 143 different “designs,” in each of two types of environment defined by the two levels of the spatial scale of the Markov random field, each replicated 25 times over the same five maps.

### Main results

For each design and each environment, we calculated the mean estimate of $\beta_{1}$ over the 25 replicates, and similarly the mean of the precision, defined to be $1/\sigma^{2}$ where σ is the SE calculated from the Hessian after estimation. Defining sample size for serially correlated data is challenging. For instance, the effective sample size in transect surveys is a number between the independent observation blocks (e.g., observation platforms or survey dates) and the total number of transect segments. Similarly, the effective sample size in telemetry studies is a value between the number of animals tagged and the fixes obtained per animal. Combining these two types of data makes the definition of sample size or information content an even less tractable problem. However, pragmatically, we can treat precision as a post hoc proxy for effective sample size.

In this simulation study, the true model is known, and the model we are fitting is essentially the same, albeit approximated as described in *Movement likelihood*. As we would therefore hope, the mean estimates were all close to the true value of $\beta_{1}$, within sampling variation for all cases and with no consistent sign in the error. In particular, the improved Langevin estimation successfully eliminates bias in these cases. The mean precision varied with the design of the experiment, naturally. To investigate the interaction between the two data types, we compared the mean precision for each design involving both telemetry and spatial survey (the majority of designs) with the corresponding values from using that amount of telemetry data and that size of survey separately. The precision from the combined analysis was always close to the sum of the precisions from the two data sources separately (within 10% in 97% of cases and within 5% in 71% of cases, with no apparent systematic differences).

For use in the comparison scenarios below, we summarized precision using a generalized additive model consisting of a linear term in the number of telemetry tracks plus a smooth term in the size of the survey area, constrained to give precision zero when both arguments were zero, that is, when there were no data.

In all cases, the precision was lower in the environments with the higher value for the spatial range of the underlying Markov random field, that is, with higher spatial autocorrelation. This reflects the fact that both telemetry and the kind of single‐region survey that we are considering involve locations that are close together spatially, so typically higher autocorrelation leads to a drop in effective sample size. However, the different structure of the data types means that the size of this drop may differ between them. This is crucial to interpreting some of the results from combined analyses below, but before exploring those in detail, it is worth pointing out some of its simpler consequences. There are a number of cases where a given number of telemetry tracks n gives a lower precision than a survey of area a when autocorrelation is high, but the same amount of telemetry gives a higher precision than the same survey when autocorrelation is low. For example, with low spatial autocorrelation, spatial range parameter 0.1, a telemetry‐only analysis with 50 tracks performed better than a survey‐only analysis with survey area of 0.5, giving a precision higher (and therefore a variance lower) by 7%. Comparing the same experiments when the spatial range parameter is 0.2 and the autocorrelation is higher reverses the inequality, with the telemetry‐only precision being lower (and the variance higher) by 15%. Thus, all other things being equal, the spatial autocorrelation in the covariate of interest can determine whether telemetry or surveying is preferable for estimating its selection coefficient, regardless of the particular form of their relative costs.

**FIGURE 1:**
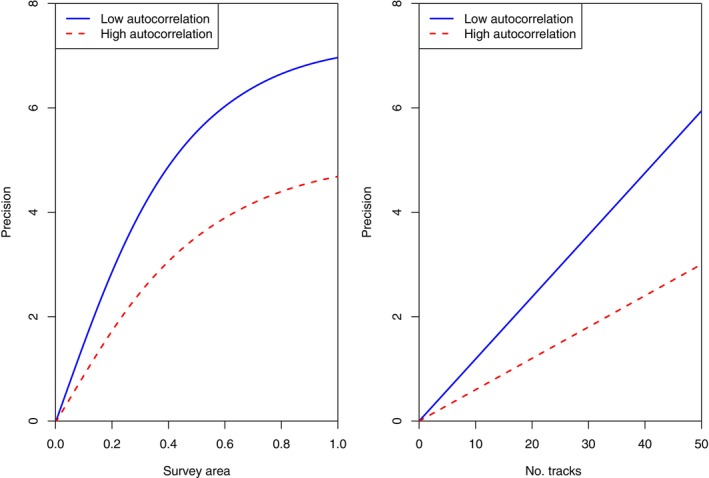
The precisions from survey‐only (left) and telemetry‐only (right) experiments, as a function of area surveyed and number of tracks, respectively, in landscapes with two different levels of spatial autocorrelation for the resource of interest.

To fully understand the performance of the integrated analysis, a key consideration is how the accuracy of estimation relates to the effort involved in data collection. Considering the data types separately, the results in our experiments show that for telemetry, the precision of estimation is very close to being linear in the number of animals tracked, as expected, and so it is reasonable to think of it as being proportional to effort. For our simple survey designs, it is natural to think of the effort involved as being proportional to the area surveyed; however, the precision increases more slowly than area, with an element of “diminishing returns.” Figure 1 shows the precision achieved by designs of each type in the two differently correlated environments.

**FIGURE 2:**
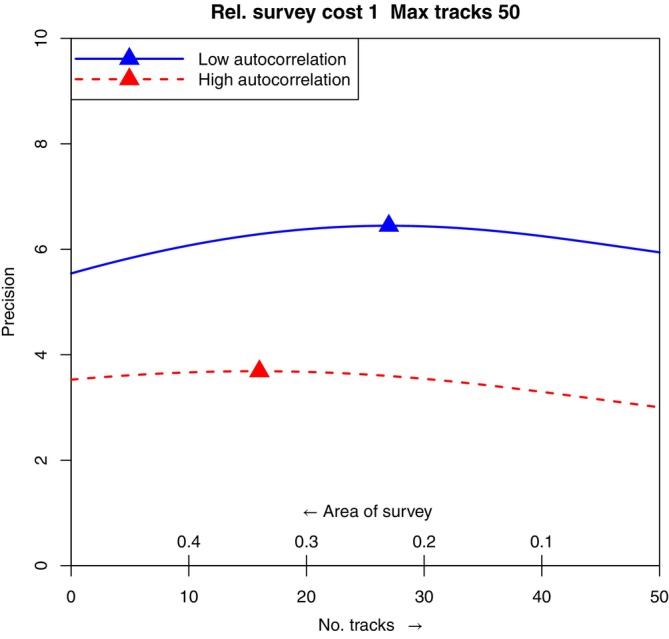
The precisions from experiments in which the allocation of a fixed total effort is varied between spatial survey only (left) and telemetry only (right), in landscapes with two different levels of spatial autocorrelation for the resource of interest. The triangles indicate the optimal allocation in each landscape.

This affects the potential choice between using telemetry, spatial survey, or both, to collect data to estimate selection, given a total amount of available effort. Without specifying a particular study area or species, it is not possible to know the relative efforts of telemetry and surveying. Instead, we choose exchange rates that represent situations where neither approach dominates completely, and look at the effect of varying the exchange rate and the total effort available. Figure 2 represents a case where the total effort is equivalent to tracking 50 animals, and shows the effect of different assumptions about how that effort is spent. The *x*‐axis indicates the effort spent on telemetry, from 0 to 50; the remaining effort (increasing from right to left) is converted into a possible size of survey area, with an exchange rate arbitrarily set to 1 in this case. The *y*‐axis then shows the precision, proportional to effective sample size, achieved for that combination of observations, based on the simulation experiments. The upper/blue solid curve corresponds to lower spatial autocorrelation (spatial range parameter 0.1) and the lower/red dashed curve to higher autocorrelation (range parameter 0.2). On each curve, the triangle indicates the optimal combination in that environment.

In these cases, the optimal allocation of effort is always a mixture of spatial survey and telemetry, but the optimal proportions differ; when SR = 0.1, the proportion is 54%, and when SR = 0.2, it is 32%. Thus, when a mixture of data types is allowed, the optimal mix can vary substantially, depending on the spatial covariance structure of the resource of interest. Figure 2 also illustrates the earlier point about survey‐only and telemetry‐only designs. The points at the left‐ and right‐hand ends of the curves represent survey‐only and telemetry‐only designs, respectively; for this combination of parameters, telemetry alone is better when autocorrelation is low, but survey alone is better when autocorrelation is high.

In the high autocorrelation case in particular, the differences in precision obtained are not all that high; their practical importance will depend on the actual costs involved and the value of precise estimation of β in a given application. Our point here is that such comparisons can be made within this framework, will sometimes favor mixed designs, and will depend on the autocorrelation in the covariate.

**FIGURE 3:**
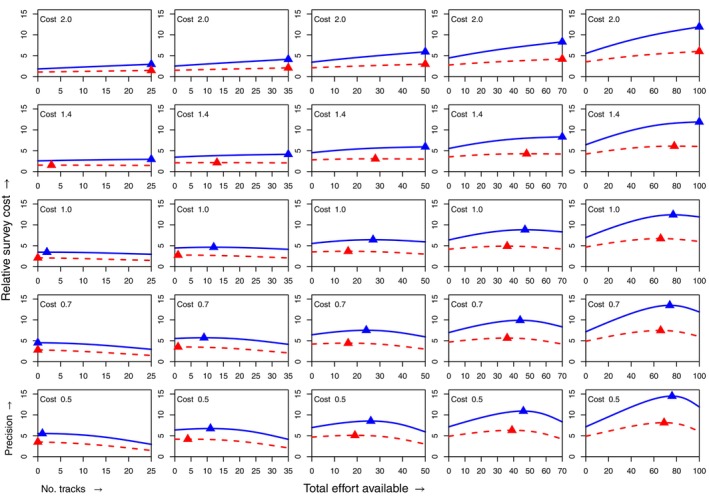
The precisions from varying the allocation of effort, for varying relative costs of spatial survey (rows; also indicated within each sub‐figure), and total amounts of effort available (columns; indicated by the range of the *x*‐axis in each sub‐figure).

Figure 3 shows the effect of varying the relative cost of surveying (rows) and the total effort available (columns). The central sub‐figure is identical to Figure 2. For the highest level shown for relative survey cost, the optimal design is always to use telemetry only; in nearly all other cases, the optimal allocation differs between the two environments with different scales of spatial autocorrelation (indicated by color and by solid/dashed lines). Where they do differ, in all cases, higher spatial autocorrelation in the resource leads to a lower proportion of effort being allocated to telemetry.

Reducing the relative cost of surveying generally *decreases* the proportion of effort that it represents in an optimal allocation, essentially due to diminishing returns. However, this effect is not universal; in some cases, the change in optimal proportion is not a monotonic function of relative cost. For example, in the column of Figure 3 representing a total available effort equivalent to tracking 35 animals (second column from the left), it can be readily seen that as the relative cost of surveying decreases (down the column), the optimal surveying proportion decreases and then increases again.

The optimal allocation also depends heavily on the total effort available, even when relative costs remain the same. For example, when the relative cost is held fixed at the same value as in Figure 2 (middle row of Figure 3), a total budget equivalent to 25 tracks should be allocated primarily on a survey (at least 90%), whereas a total budget equivalent to 100 tracks should be allocated mainly to telemetry (at least 60%).

Again, the magnitude of these differences varies, and their practical importance will be a function of factors outside the scope of this study. Our simulations show, however, that a mixture of telemetry and spatial survey can be optimal over a wide range of scenarios and that the optimal design depends in a complex way on relative costs, available effort, and the spatial pattern of the resource.

### The effect of $G\left(\cdot \right)$

For the results in *Main results*, the distribution $G\left(\cdot \right)$ used to improve the Langevin approximation always takes the same form, described in detail in *Inference*, since empirically that reduces the bias caused by the approximation. In this section, we look more closely at this effect in a particular case, and briefly consider other possible choices of $G\left(\cdot \right)$. Obviously, the choice of $G\left(\cdot \right)$ affects only the inference from the telemetry, so in this section we consider telemetry only.

**FIGURE 4:**
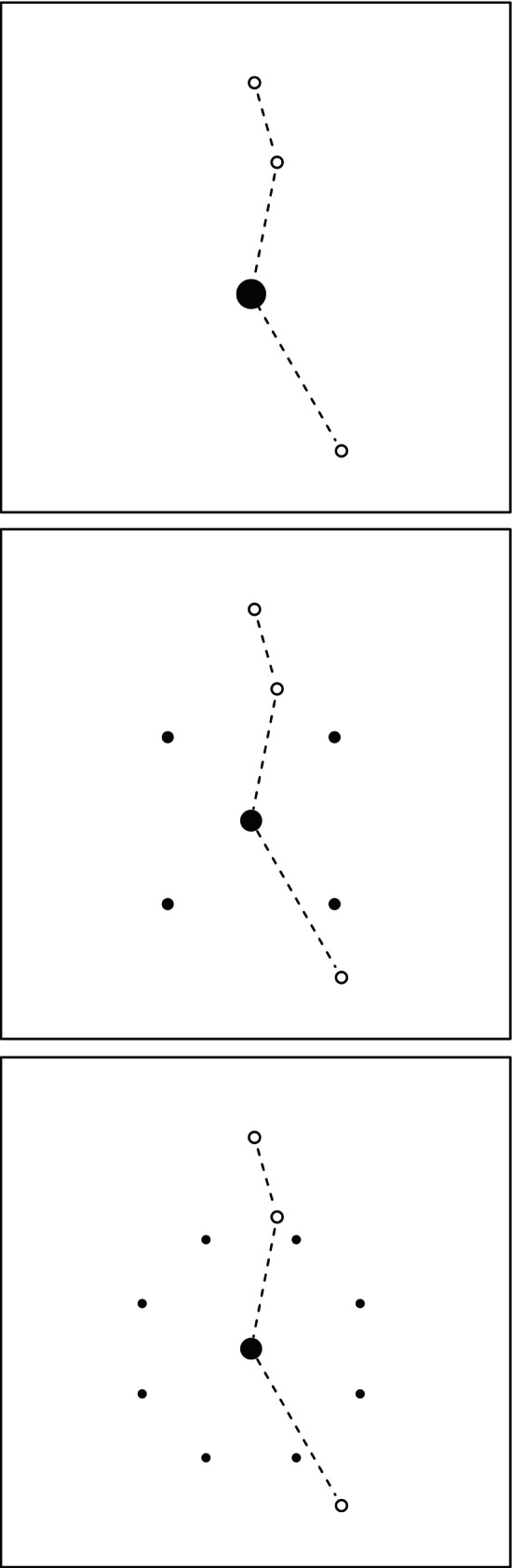
Illustration of the different methods for smoothing the gradient of the target density when analyzing telemetry within the Langevin model. The dashed lines link successive observations (open circles), shown for scale. The solid disks show the locations at which the gradient is evaluated when calculating the Langevin drift term for a step starting at the central (or only) such point; the areas of the disks are proportional to the weights used in calculating the average gradient.

We focus on the case covered in Figure 2, with spatial range parameter 0.1, that is, a “low autocorrelation” environment as described above. The 25 simulated datasets each of 50 telemetry tracks, across five maps, are each analyzed in three ways: using the unsmoothed gradient function $b\left(\cdot \right)$; using the smoothed version $\tilde{b}\left(\cdot \right)$ as in the main simulation experiment, with four additional points; and using a potentially better approximation based on eight additional points, again equidistant from the starting point x; see Figure 4.

In each smoothed case, the gradient is a weighted average, with weight one‐half at the central point, and the remaining weight split equally between the outer points. The estimated biases are −0.302 for the unsmoothed method, −0.017 with four additional points, and −0.023 with eight additional points. This illustrates the general result found that smoothing with a simple choice of $G\left(\cdot \right)$ essentially eliminates bias in the cases considered and that additional computational effort in the smoothing does not necessarily give any further improvement.

It is also informative to look at the individual estimates obtained using these different forms for $G\left(\cdot \right)$ with the same datasets.

**FIGURE 5:**
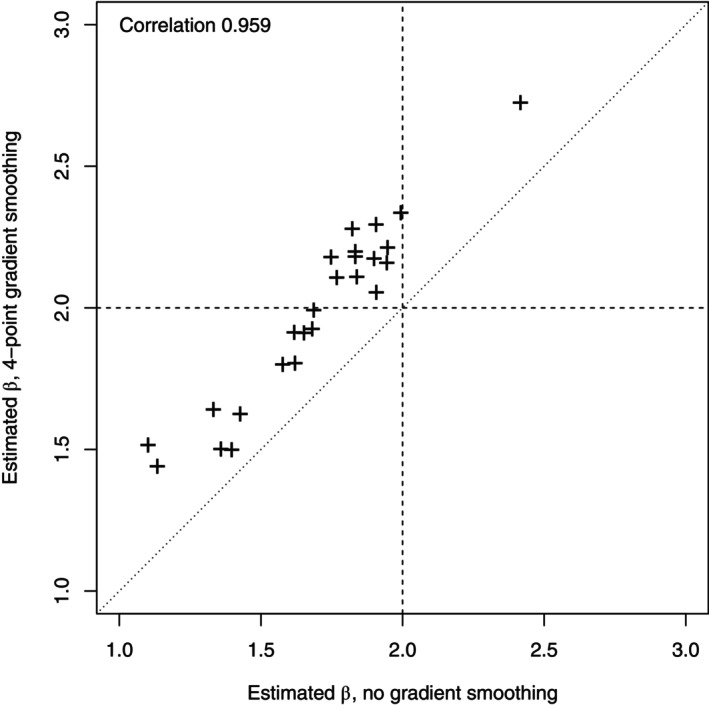
The relationship between estimates using smoothed and unsmoothed gradients. The horizontal and vertical dashed lines indicate the true value of $\beta_{1}$; the diagonal dotted line is the line y=x, so points on that line would indicate identical estimates from each method.

Figure 5 shows the estimates from the naive method using $b\left(\cdot \right)$ plotted against those from the method of *Inference*, using $\tilde{b}\left(\cdot \right)$ with four additional points. Each point in the plot represents a single simulated dataset. The estimates have very high correlation (0.959), showing that this smoothing produces estimates of $\beta_{1}$ that are very similar to the unsmoothed method except that they are shifted so as to reduce the bias; the smoothing addresses the bias but does not reduce the variability in the estimates. A similar comparison (not shown) between the two smoothed versions above, with four and eight additional points, shows that the individual estimates are always very similar, with extremely high correlation (0.986) and no systematic differences, confirming that the additional smoothing of $\tilde{b}\left(\cdot \right)$ leaves estimates essentially unchanged.

The choice of $G\left(\cdot \right)$ is further considered in the *Discussion*.

### Case study

**FIGURE 6:**
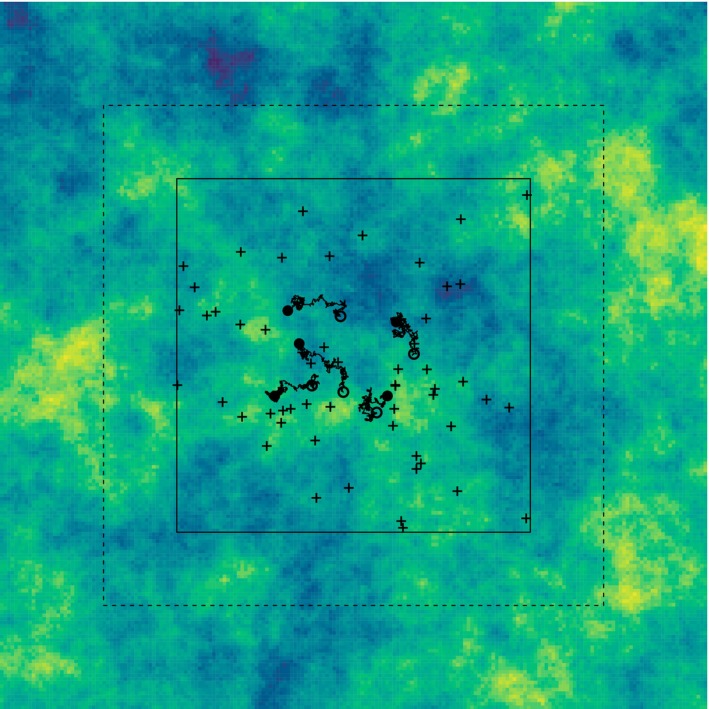
The simulated resource map used for the case study. Also shown are potential small (solid line) and large (dashed line) survey regions, the observations from the smaller survey (+), and five of the telemetry tracks (starting at the open circles and ending at the solid disks).

To illustrate our approach, we have added a simulated case study, looking in more detail at a particular situation. We use a single (simulated) resource map, as shown in Figure 6.

**FIGURE 7:**
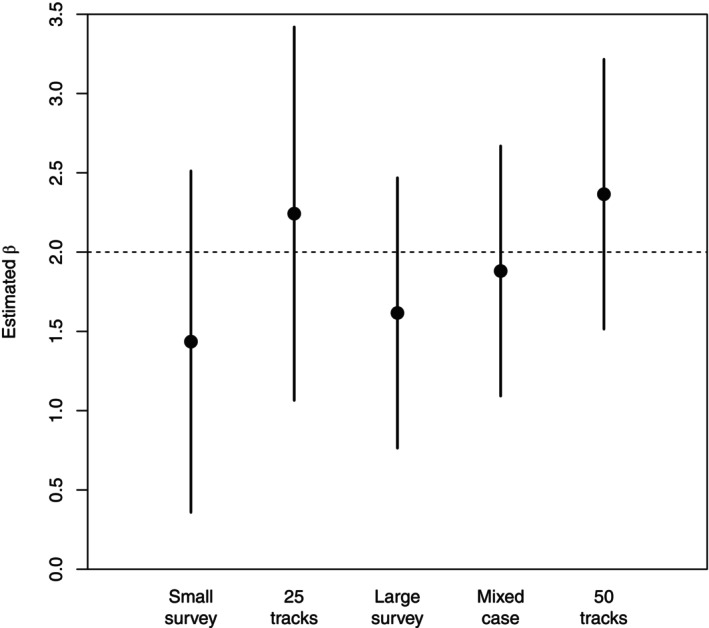
The estimates of $\beta_{1}$ and corresponding Hessian‐based 2 SD intervals for different amounts and allocations of sampling effort in the case study.

The region shown is the square $\left[-1/2,1/2\right]\times \left[-1/2,1/2\right]$; the actual simulated region is $\left[-1,1\right]\times \left[-1,1\right]$, as above, to accommodate rare individual excursions. A spatial range of 0.1 is used in generating the resource surface; for simplicity, all other parameters, including those for localization, are kept the same as in the main simulation study. We consider the quality of estimation of a single selection parameter $\beta_{1}$, with the effort available equivalent to 50 telemetry tracks of a given duration, or a single spatial survey (“larger”; dashed lines in the figure), or a combination of 25 tracks and a survey covering half the area of the larger one (“smaller”; solid lines in the figure). To assist visualization, the results of the smaller survey are also shown in the figure, as are a subsample of the telemetry data. The results of *Main results* suggest that the mixed design will on average perform best, and that is in fact the case for this realization. Figure 7 shows the estimates of $\beta_{1}$ for each design, along with approximate 2‐SD intervals. These intervals are based on the numerically obtained Hessian in each case, rather than on the results from the simulation study. The results of the two “sub‐designs” (smaller survey only, or 25 tracks only) are also shown for comparison, and the true value of $\beta_{1}$ is indicated by the dashed line. The benefit of the mixed design over the other two designs of nominally equal effort is modest, as expected from the main simulation results; in this case, underestimation of $\beta_{1}$ by the spatial surveys is balanced by overestimation by the telemetry, but this is case‐specific, rather than indicative of true bias.

## DISCUSSION

Our first aim in this paper was to derive a joint likelihood for the integrated and efficient analysis of telemetry and spatial survey data. We used recent statistical results (Michelot, Blackwell, Chamaillé‐Jammes, et al., 2019; Michelot, Blackwell, & Matthiopoulos, 2019; Michelot, Gloaguen, et al., 2019) that make the connection between HSFs (the broad class of approaches used for animal distribution modeling) and SSFs (the class of models used for the statistical analysis of habitat preference from telemetry data). These methods employ individual movement models that ensure the correct scaling of stepwise habitat selection by many individuals to their long‐term utilization distributions or to the emergent utilization distribution of the population to which they belong.

The movement model used here, the Langevin diffusion, is perhaps the simplest continuous‐time model for which short‐term and long‐term behavior can be related parametrically. Our general framework, however, can be applied using any movement model for which that parametric link can be made, and expanding the range of such models is an active area of research.

The likelihood function derived on this basis can be implemented in any inferential framework, whether frequentist or Bayesian, and the resulting computation is exactly as fast as traditional computation using each data type alone. Indeed, by allowing the computation of covariate gradients prior to the main analysis, our approach to telemetry data may prove to be faster than some implementations of SSFs via conditional logistic regression, that require the use of “available” control points (Thurfjell et al., 2014), and hence may inflate the analysis data frames by one or more orders of magnitude. Future investigations on computational gains are particularly pertinent to telemetry datasets which are currently swelling to terabyte levels (Nathan et al., 2022).

Our implementation of the method here was based on simple maximization of the likelihood. However, the fast evaluation of the likelihood and its derivatives means that it would be straightforward to combine this modeling framework with off‐the‐shelf Markov chain Monte Carlo inference for more detailed analysis.

Our strategy for minimizing bias in the use of the Langevin model depends on the choice of a local distribution $G\;\left(z|x,\zeta \sqrt{\mathrm{\delta t}}\right)$, centered at x and with scale parameter $\zeta \sqrt{\mathrm{\delta t}}$, used to better approximate the gradient terms for a step originating at x over an interval of duration δt by averaging gradients from locations z. Our simulation results show that often good performance comes from a simple, discrete choice for $G\left(\cdot \right)$, with ζ derived directly from the telemetry data rather than estimated jointly with the model parameters. In principle, the optimal ζ is likely to be related to the movement parameter γ; however, γ is sufficiently well estimated, and the bias‐reduction sufficiently robust, that this seems unnecessary. Similarly, in principle the calculated gradient term $\tilde{b}\left(x\right)$ should reflect the whole neighborhood of x and so $G\left(\cdot \right)$ ought to be a continuous distribution, such as a bivariate normal distribution integrating over all locations near x, or even a distribution that itself incorporates values of the covariates. Empirically, however, this seems unlikely to give any practical improvement. Using a discrete $G\left(\cdot \right)$ and fixing ζ before the optimization of the parameters—α and β in particular—allows much faster computation, since the gradients of the covariates in the calculation of $\tilde{b}\left(x\right)$ can be calculated just once. Of course, if the speed of movement or the time intervals between observations are large enough, the approximation to the Langevin process will break down, and there may be intermediate cases where more sophisticated choices of $G\left(\cdot \right)$ will matter. Conversely, shorter time intervals (i.e., smaller δt) will lead to better approximation to the Langevin process. Note, however, that for simulation experiments, the time step in the simulation needs to be much smaller than δt to avoid the approximation in simulating the continuous‐time Langevin process becoming a concern in its own right.

Allowing the calculation of $\tilde{b}\left(x_{j}\right)$ to depend on the following observation $x_{j+1}$ seems appealing, but since the need is to calculate a distribution for $x_{j+1}$ given $x_{j}$, for example as in Equation (3), to do so would involve a corresponding calculation for the range of potential values of $x_{j+1}$. An approach of this kind is possible in principle, but it would be closer in spirit, and in computational cost, to the methods that partially reconstruct the trajectory between observations, mentioned in *Movement likelihood*.

By allowing the estimation of mobility parameters simultaneously with selection parameters, our approach retains features of best practice, as stipulated in the integrated SSF literature (Avgar et al., 2016; Forester et al., 2014). This statistical link between individual movement and species distribution models will ultimately allow analysts to ground‐truth findings at any organizational level of interest using knowledge acquired at another level. For instance, we envisage Bayesian inference approaches at population level being bolstered by fundamental information shaping the priors of individual mobility and behavior.

Having shown that joint inference between spatial survey and telemetry data is possible and efficient, our second objective was to generate some intuition about its statistical performance (precision of estimation of underlying parameters). Our simulation results suggest that, as might have been expected, pooling data leads to higher precision, but more importantly, even if the amount of effort is kept fixed, the collection of both types of data is advisable in many cases, often over a range of values for their relative costs.

Spatial surveys provide information based on where animals are not observed, as well as where they are; consequently, they have the potential to span areas that are contrastingly different in terms of their habitat suitability for the species. This enables the model to detect broad trends in habitat preference. On the other hand, telemetry data follow the finer scale decisions of the animals and can therefore provide species‐relevant stratification to the dataset. These differences between the way space is sampled have previously meant that the predictions of HSFs and SSFs had not been compatible, but by developing a likelihood that ensures their compatibility, we have been able to address this problem and combine the strengths of each approach.

The results shown here are unavoidably dependent on specific assumptions about the environment, behavior, and data collection incorporated in the simulation experiments. There will of course be situations where one approach to data collection dominates the other, for reasons of feasibility or cost, however measured. Our analytical results show how to perform a joint analysis of the two key data types in animal spatial ecology. Our simulation results indicate that a mixed analysis can be optimal in a wide range of cases and that the optimal design can depend on variables not always considered in current practice, such as the level of autocorrelation in relevant environmental variables.

In our simulation study, we have focused on estimation of the selection parameter for a measured spatial covariate, as is standard in habitat selection and step selection. We have included some types of localization within the same framework, for example, by defining attraction to an unknown central place through latent covariates with unknown but estimable coefficients, but it is important to realize that if such terms were of primary interest, then the optimal balance of sampling effort would change. For example, such covariates defining central place attraction may well change slowly in space compared with other covariates, in which case a desire to estimate their coefficients well would typically increase the value of spatial surveys covering larger or more widespread areas. This can be seen as a further example of our result that the spatial autocorrelation of covariates is crucial in designing data collection schemes. Detailed investigation is beyond the scope of this paper, but would certainly benefit from the methodology introduced here.

The bias in the estimation of β from telemetry using the Langevin method of Michelot, Gloaguen, et al. (2019), without our improvement of *Movement likelihood*, depends on (among other things) the time interval δt between observations. In the cases we have analyzed in detail, our improvement appears to completely eliminate bias. However, as already mentioned when considering the choice of $G\left(\cdot \right)$, in less favorable cases, a more elaborate $G\left(\cdot \right)$ may be needed. It is likely that in such cases, a smaller δt will also help to reduce or eliminate bias. This is an argument in favor of higher frequency collection of telemetry data in cases where the estimation of β is difficult, for example, because of rapid movement or high spatial autocorrelation of resources. As well as applying to the current Langevin model, we conjecture that the same would apply to any approach that models selection coherently in continuous time.

Our method relies on the key assumption that the individual movement models embedded in our joint likelihood are consistent with the steady‐state distribution implied by our population utilization models. The mathematical theory underpinning Michelot, Gloaguen, et al. (2019) shows that this will hold provided that the telemetry and survey data are sampling statistically identical populations, environmental spaces, and temporal frames (both diurnal and seasonal). In some applications, these assumptions may not hold (Carroll et al., 2019; Phillips et al., 2019; Sansom et al., 2018). For example, it is possible that particular classes of animals (e.g., different ages or sexes) are easier to capture for tagging or that tagged animals go to different habitats than those visited by surveys. Temporal imbalances may also be influential (Carroll et al., 2019). It is also possible that surveys happen at different times of the year than tagging effort when animals are performing different life history functions or that, unlike satellite fixes, visual observations cannot be delivered at nighttime. Of course, all these considerations limit the interpretation of data of these kinds even when considered separately. If, for example, spatial surveys happen only at one time of year, they will give information about distribution at that time in a relatively straightforward way, but will not be generalizable to other times of year without additional assumptions or information. Furthermore, in most existing comparisons, any consideration of the relationship between short‐term and long‐term distributions is ignored. So these limitations are not specific to our approach; rather, the desire to combine datasets may highlight the possibilities for, and constraints on, generalizability. To address such constraints, extending the methodology to accommodate such differences is current work in progress.

The parameter α, which simply scales the overall rate of observations in the spatial survey, depends on population size, observability of individuals, and the intensity or effort in the survey. We treat it here as a nuisance parameter, but it should be stressed that its value will affect the information obtained from any spatial survey, and hence the optimal distribution of effort between types of data collection. For example, all other things being equal, lower survey counts due to low population or low observability will make spatial surveys less informative for a given expenditure of effort. We have not investigated the effect of α quantitatively, but it is likely that it will have a qualitatively similar effect to changing the relative cost of survey and telemetry, that is, moving vertically within Figure 3.

For a spatial survey covering a very small area, the variation in the number of individuals observed means that the actual precision of estimation varies enormously, particularly if there is an appreciable probability of observing no individuals at all. The summaries in *Main results* are based on means over maps and replicates. In cases where the total effort available is rather smaller than any of those in Figure 3, this unpredictability may be an additional factor in favor of telemetry. It would make sense to consider a criterion for optimization that was more nuanced than simply minimizing expected variance. We have not explored such scenarios in detail, but our framework certainly facilitates such experiments.

The initial observation in telemetry *may* also be an observation from (or at least related to) the target distribution. In such cases, rather than conditioning on the initial location, we can incorporate it into the analysis. If it comes directly from the target distribution, as is sometimes the case where telemetry arises in bursts, it can be very informative (Blackwell, 2003; Dunn & Gipson, 1977) flexible way. Similarly, detection by spatial survey may rely on the individuals having been tagged first (Melnychuk & Christensen, 2009), leading naturally to a need to combine the different data types. The inconsistencies between HSFs and SSFs lead to difficulties in interpretation if existing methods are used, but our development of a parameterization and likelihood that ensures their compatibility means that this potential problem becomes an inferential opportunity.

The models and results presented here give a coherent way to combine telemetry and spatial survey data and give insight into the trade‐offs between them and how they are affected by spatial pattern of resources and other covariates. Current work involves extending this conceptual framework to a wider range of movement models and survey types so that considering these two fundamental data types jointly can become the norm, both in the planning of experiments and in their analysis.

## AUTHOR CONTRIBUTIONS

Paul G. Blackwell and Jason Matthiopoulos conceived and developed the methodology, investigated its properties, and wrote the paper. Paul G. Blackwell implemented the methods and carried out the simulations.

## CONFLICT OF INTEREST STATEMENT

The authors declare no conflicts of interest.

## Data Availability

Code (Blackwell, 2023) is available in ORDA, the University of Sheffield's online research data repository, at https://doi.org/10.15131/shef.data.23708286.v1.
